# Effects of Re-Vitrification of Mouse Morula and Early Blastocyst Stages on Apoptotic Gene Expression and Developmental Potential

**DOI:** 10.22074/cellj.2018.4892

**Published:** 2017-11-04

**Authors:** Nasrin Majidi Gharenaz, Mansoureh Movahedin, Zohreh Mazaheri

**Affiliations:** Department of Anatomical Sciences, Faculty of Medical Sciences, Tarbiat Modares University, Tehran, Iran

**Keywords:** Apoptosis, Embryo, Genes, Mouse

## Abstract

**Objective:**

Re-vitrification of embryos immediately after thawing or after a culture period could be used to preserve the extra embryos after embryo transfer. This study aims to clarify the effect of re-vitrification on *Bax* and *Bcl-2* gene expressions of compact and early blastocyst stage embryos.

**Materials and Methods:**

This experimental study was performed on mouse embryos. We collected 8-cell stage embryos (n=400) from female mature mice, 60-62 hoursafter injection of human chorionic gonadotropin (hCG). The embryos were divided into 5 groups: fresh (n=80), vitrified at the 8-cell stage (n=80), vitrified at the blastocyst stage (n=80), vitrified at the 8-cell stage, and re-vitrified at the compact (n=80) and early blastocyst stages (n=80). Embryos were vitrified by cryolock. We analyzed the developmental rates of the vitrified-warmed embryos with the chi-square test. Quantitative polymerase chain reaction (qPCR) data were analyzed with SPSS version 16 using one-way analysis of variance (ANOVA) followed by Tukey’s post hoc test. P<0.05 were considered statistically significant.

**Results:**

The expanded blastocyst formation rate showed a significant difference in re-vitrified embryos compared with fresh embryos (P<0.05). However, this result was similar between the two re-vitrified groups. Our data showed a significant difference in expression of the *Bax* and *Bcl-2* genes between re-vitrified and fresh embryos (P<0.05). Expressions of the *Bax* and *Bcl-2* genes showed no significant difference between the two re-vitrified groups.

**Conclusion:**

Based on our study, re-vitrification could affect developmental rate and expressions of the *Bax* and *Bcl2* genes.

## Introduction

Cryopreservation of embryos leads to increased cumulative pregnancy rates along with decreased costs of artificial reproductive technology and reduces the risks for ovarian hyperstimulation syndrome and risks of multiple pregnancy, as well as preservation of fertility for patients who undergo chemotherapy and radiotherapy (1, 2). Re-cryopreservation of the same embryos after thawing has both theoretical and applied cryobiological implications (3). Occasionally, unexpected extra embryos are available after thawing. Hence, re-cryopreservation after frozen-thawed embryo transfer is an option. The potential to refreeze previously frozen embryo samples would be useful should an ampule be thawed that contained more embryos than required for transfer (4). In assisted reproduction technology, the chief adverse treatment outcome is the high incidence of multiple pregnancies (5).

Researchers recommend selective single embryo transfer (eSET) to avoid multiple pregnancy and sparse embryos could be cryopreserved. Although cryopreservation of mammalian embryos is now a routine method, considerable differences in efficiency exist depending on the embryo stage, species, and origin (6). Some researchers have reported that the developmental stage of an embryo during vitrification has an important role in survival rate after the vitrification process (7, 8). Han et al. (9) cryopreserved rat embryos at various developmental stages and reported that the best stage for vitrification was the morula stage. However other researchers reported that the morula stage was more sensitive to cryopreservation compared to blastocysts. Hence, these researchers have stated that the best developmental stage for vitrification was the blastocyst stage. Blastocysts have the advantage of passing genomic activation and contain numerous small cells; thus, the loss of some cells during vitrification and warming might be less damaging for further embryo development (10, 11). We have chosen the compact and blastocyst stages to compare the effects of re-vitrification on various embryo developmental stages.

Researchers have reported that double conventional freezing of mice and human embryos, followed by re-vitrification of bovine oocytes and expanded blastocysts with different vitrification techniques resulted in the birth of offspring (3). However there is inadequate data on the repeated use of vitrification. Understanding the reasons and mechanisms for injury may help to select the best embryo developmental stage and cryopreservation methods to avoid toxic or irreversible damages. Embryo and oocyte cooling possibly cause a risk for intracellular ice formation, uncontrolled dehydration, and increased viscosity which may be lethal to the oocytes and result in embryo injury. In addition, re-crystallization and osmotic shock could happen during the thawing process and possibly reduce survival rates (2, 12). A profile of the apoptotic genes can be affected by the vitrification procedure. Apoptosis is a common form of cell death regulated by members of the Bcl-2 family. The Bcl-2 family is subdivided into two major groups, pro-apoptotic (*Bax*) and anti-apoptotic (*Bcl- 2*). In healthy cells, *Bax* localizes within the cell cytoplasm. During apoptosis, *Bax* is translocated from the cytosol to the mitochondria. Pro-apoptotic factors, such as cytochrome c, are released from the mitochondria, which can trigger the caspase pathway and lead to cell death. Bcl-2 is located in the outer mitochondrial membrane and prevents apoptotic mitochondrial change (13). The current study investigates the effects of revitrification during various embryo stages on developmental rate, in addition to expressions of *Bax* and *Bcl-2*.

## Materials and Methods

We used mouse embryos for this experimental study. Female NMRI mice (6-8 weeks) were purchased from Pasteur Institute (Iran). The mice (n=20) were housed in a room under standard laboratory conditions (12 hour light, 12 hour dark at 22˚C) with free access to water and standard food. The Tarbiat Modares University Ethical Committee approved this study. All procedures were performed according to the protocol approved by the Committee of Medical Sciences Faculty (59/4978) at Tarbiat Modares University.

### Embryo collection and culture

**Fig.1:**
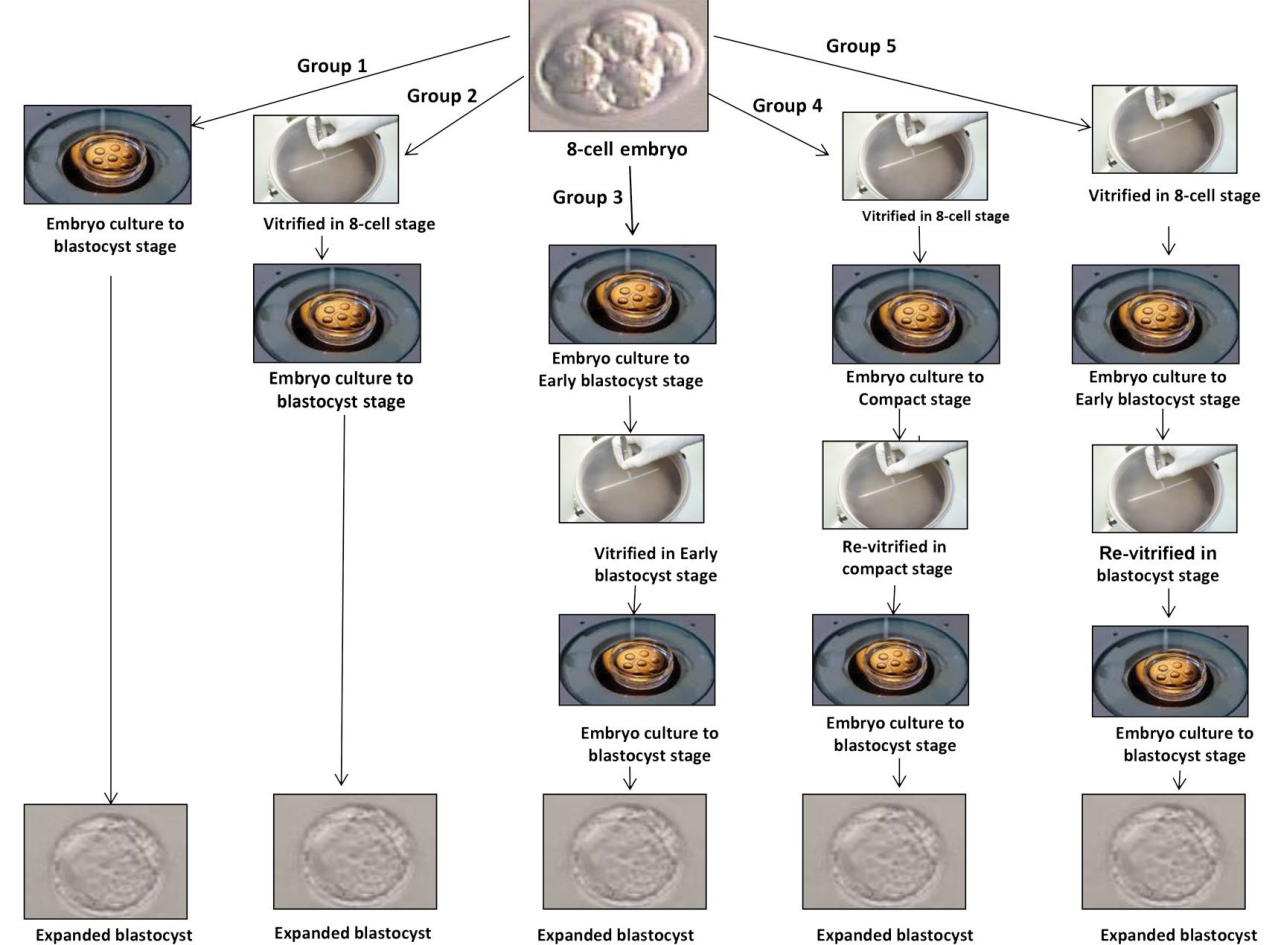
Schematic picture represents the re-vitrification process in different developmental stages. Group 1; Fresh embryos, Group 2; 8-cell vitrified embryos, Group 3; Early blastocyst stage vitrified embryos, Group 4; Vitrified at the 8-cell stage and re-vitrified at the compact stage, and Group 5; Vitrified at the 8-cell stage and re-vitrified at the early blastocyst stage.

Mice were superovulated by an intraperitoneal (IP) injection of 7.5 IU pregnant mare serum gonadotropin (PMSG, Intervet, Inc., The Netherlands) followed 48 hours later by 7.5 IU human chorionic gonadotropin (hCG, Pregnil, Organon, Oss, The Netherlands).The female mice were mated with a single male of the same age (6-8 weeks old). Mating was confirmed by an examination for the presence of a vaginal plaque in the female mice. Mice that had vaginal plaque were considered to be pregnant. Pregnant female mice were sacrificed by cervical dislocation 60-62 hours after the hCG injection in order to collect the 8-cell embryos (14). The 400 collected embryos were divided in to five groups: i. 8-cell embryos cultured in human tubal fluid (HTF, Geneocell ideal, Iran) medium supplemented with 10% human serum albumin (HSA, Biotest, Germany) until 72 hours (n=80, group 1), ii. 8-cell embryos vitrified-warmed for 2 hours, then cultured until 72 hours (n=80, group 2), iii. 8-cell embryos initially cultured until 30-36 hours, then vitrified-warmed at the early blastocyst stage, and cultured until 24-30 hours (n=80, group 3), iv. 8-cell embryos were vitrified-warmed and after 6-8 hours, the alive embryos were re-vitrified at the compact stage. Then, compact embryos were cultured until 64 hours (n=80, group 4), and v. 8-cell embryos were initially vitrified-warmed. After 30-36 hours, the alive embryos were re-vitrified at the early blastocyst stage and then cultured until 36 hours (n=80, group 5, Fig .1).

### Vitrification procedure

Mouse embryos were vitrified by a two -step procedure with a vitrification kit (Geneocell ideal, Iran) using cryolock (Biotech, USA) as a carrier according to Kuwayama et al. (15). Embryos were initially equilibrated in equilibration solution (ES) at room temperature for 15 minutes. The ES consisted of 7.5% (v/v) ethylene glycol (EG, Sigma Germany) and 7.5% (v/v) dimethyl sulfoxide (DMSO, Sigma Germany) dissolved in HTF medium supplemented with 10% HSA. After initial shrinkage, the embryos regained their original volume and were transferred to vitrification solution (VS) for 1 minute. The VS consisted of 15% (v/v) EG, 15% (v/v) DMSO, and 0.5 mol/L sucrose (Sigma, Germany) dissolved in HTF medium supplemented with 10% HSA Within less than 60 seconds, two to three embryos in minimal VS (<1.0 μl) were placed onto the inner surface of the cryolock carrier. The cryolock was subsequently plunged vertically into liquid nitrogen.

### Warming technique

After the cryostorage, we warmed the embryos using a three-step dilution procedure with a vitrification kit (Geneocell ideal, Iran). Briefly, the cryolocks which contained the embryos were removed from liquid nitrogen and dipped into T1 solution (Geneocell ideal, Iran) that consisted of 1.0 mol/L sucrose at 37˚C. After a 1 minute equilibration in T1, the embryos were placed into T2 solution (37˚C) that contained 0.5 M sucrose for 3 minutes. Then, the embryos were placed into T3 solution (37˚C) that contained 0.25 M sucrose for 3 minutes. Finally, the embryos were washed with HTF medium.

### Assessment of embryo survival

We determined the viability of the warmed embryos based on visual examination of the integrity of embryo membrane, zona pellucida, and normality of the cytoplasm at two hours after warming. During the in vitro culture, embryo development was evaluated daily. The rate of blastocyst formation was evaluated by the numbers of late blastocysts to survived embryos post-vitrification.

### Molecular assessment by quantitative polymerase chain reaction

We extracted total RNA from the embryos in each group (n=25) using QIAzol (Qiagen Germany) according to the manufacturer’s recommendations. In order to eliminate genomic DNA contamination, RNA samples were treated with DNase I using a kit (EN0521, Fermentas, Germany). The RNA concentration was determined by spectrophotometry. The RNA samples were stored at -80˚C until use. cDNAs were synthesized in a total volume of 20 μl that contained 5 μg total RNA either with reverse transcriptase (+RT cDNA) or without the enzyme (RT control) using a cDNA kit (Fermentas, Germany) and stored at -20˚C until use. All experiments were carried out in triplicate. For polymerase chain reaction (PCR), primers for the *Bax* and *Bcl-2* genes were designed by a NCBI website and Gene Runner software, and synthesized by Cinnagen (Iran, Table 1). PCRs were performed using Master Mix and SYBR Green in a StepOne™ thermal cycler (Applied Biosystems, USA). The PCR program began with an initial melting cycle for 5 minutes at 95˚C to activate the polymerase, followed by 40 cycles of melting (15 seconds at 95˚C), annealing (30 seconds at 58˚C), and extension (15 seconds at 72˚C). The quality of the PCR reactions was confirmed by melting curve analyses. Efficiency was determined for each gene using a standard curve (logarithmic dilution series). For each sample, the reference gene (GAPDH) and target gene were amplified in the same run. The reference gene was approximately equal. The target genes were normalized to a reference gene and expressed relative to a calibrator (group 1 as the control group).

### Statistical analysis

We used the chi-square test to analyze the blastocyst developmental rates. All embryos were randomly divided into three control and two experimental groups. The experiments were performed in triplicate. Quantitive PCR (qPCR) data were presented as mean ± SD and analyzed by SPSS software (version 16.0, Chicago, IL, USA) using one-way analysis of variance (ANOVA) followed by Tukey’s post hoc test. P<0.05 was considered statistically significant.

**Table 1:** Genes, primers, and amplification products (base pairs) for quantification of gene expression by real-time quantitative polymerase chain reaction (qPCR)


Gene	Primer sequence (5ˊ-3ˊ)	Length	Code number	Temperature

*GAPDH*	F: GACTTCAACAGCAACTCCCAC	125	NM_001289726.1	80
	R: TCCACCACCCTGTTGCTGTA			
*Bax*	F: CGGCGAATTGGAGATGAACTG	161	XM_006540584.1	83.5
	R: GCAAAGTAGAAGAGGGCAA			
*Bcl2*	F: ACCGTCGTGACTTCGCAGAG	239	NM_009741.1	84
	R: GGTGTGCAGATGCCGGTTCA			


## Results

### Survival rate in the experimental groups

A significant difference existed between the revitrification compact (87.5%) and early blastocyst (84.5%) stages compared to fresh embryos in terms of survival rate (P<0.05).There was no significant difference in survival rates of vitrified embryos at the 8-cell (88.8%) and early blastocyst (92.2%) stages compared to the revitrified embryos from the compact (87.5%) and early blastocyst (84.5%) stages. There was a similarsurvival rate for embryos in the re-vitrified groups-compact (87.5%) and early blastocyst (84.5%) stages (Table 2).

### Development to expanded blastocyst

Based on the current findings, the expanded blastocyst formation rate significantly decreased (P<0.05) in re-vitrified embryos at the compact (82.9%) and early blastocyst (81.7%) stages compared to fresh embryos (92.5%). However, we observed no significant difference inexpanded blastocyst formation rate of vitrified embryos with revitrified embryos. There was no significant difference in revitrified embryos at the compact and early blastocyst stages in terms of blastocyst formation rate (Table 2).

### Investigation of degeneration rates

The degeneration rate showed significant differences between the re-vitrified compact (17.1%) and early blastocyst (18.3%) stages compared to fresh embryos (7.5%, P<0.05). There was no significant difference between the re-vitrified compact (17.1%) and early blastocyst (18.3%) stages and vitrified 8-cell (11.2%) and early blastocyst (12.5%) stage embryos. There was a similar embryo degeneration rate between the two revitrified groups (Table 2).

### Gene expression profile

**Fig.2:**
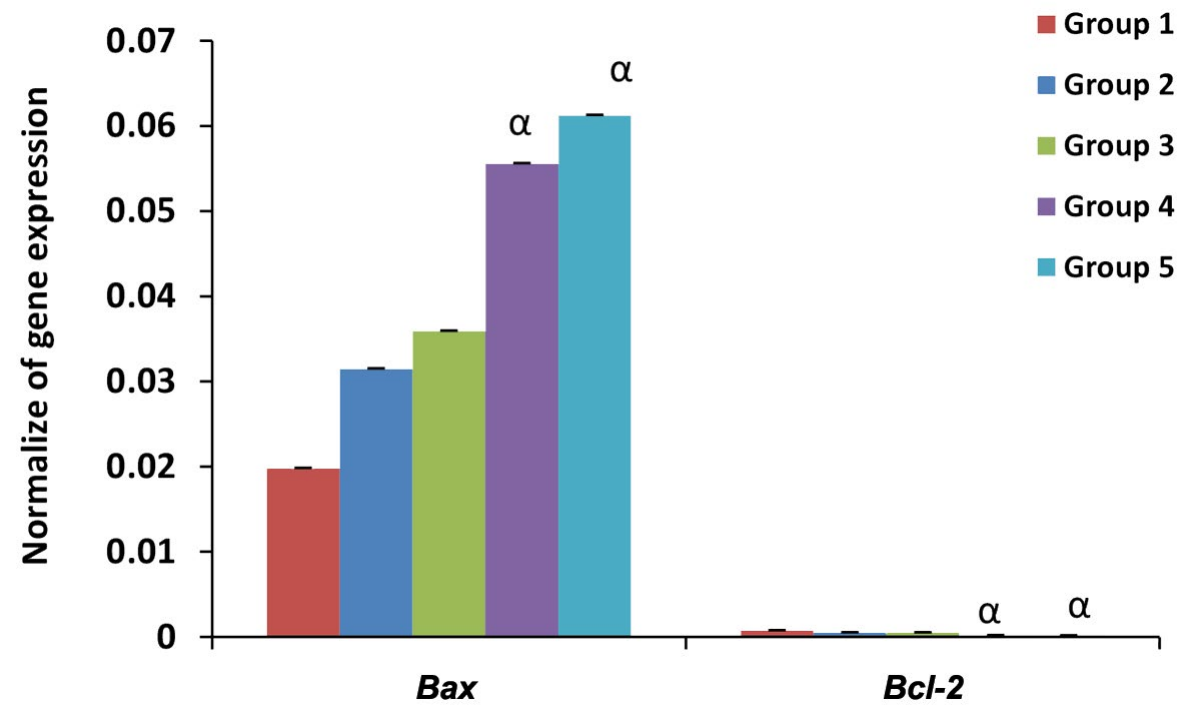
Profile of apoptotic gene expressions. Group 1; Fresh embryos, Group 2; 8-cell vitrified embryos, Group 3; Early blastocyst stage vitrified embryos, Group 4; Vitrified at the 8-cell stage and re-vitrified at the compact stage, Group 5; Vitrified at the 8-cell stage and re-vitrified at the early blastocyst stage, and α;Significant difference with the control 1 (P<0.05).

*Bax* had a significantly (P<0.05) higher expression in re-vitrified compared to fresh embryos. In contrast, *Bcl-2* had significantly (P<0.05) less expression in re-vitrified compared to fresh embryos. The re-vitrification procedure upregulated *Bax* expression (pro-apoptotic gene) and downregulated *Bcl-2* expression (anti-apoptotic gene) in the re-vitrified embryos. In this regard, no significant difference existed between re-vitrified and vitrified embryos. Both *Bax* and *Bcl-2* had similar expressions between re-vitrified embryos at the compact stage and early blastocyst stage (Fig .2).

**Table 2:** Embryonic developmental in the experimental group


Group	Fresh embryos(n)	Vitrified embryos(n)	Survival rate of vitrified embryos(%)	Re-vitrified embryos	Survival rate of re-vitrifiedembryos(%)	Expanded blastocystsn (%)	Total degeneration n (%)

1	80	-	-	-	-	74(92.5)	6(7.5)
2	80	80	71(88.8^a^)	-	-	71(88.8)	9(11.2)
3	80	80	74(92.2^a^)	-	-	70(87.5)	(10) (12.5)
4	80	80	72(90^a^)	72	70(87.5^a^)	66(82.9^a^)	14(17.1^a^)
5	80	80	69(87^a^)	69	67(83.75^a^)	65(81.7^a^)	15(18.3 ^a^)


Group 1; Fresh embryos, Group 2; 8-cell vitrified embryos, Group 3; Early blastocyst stage vitrified embryos, Group 4; Vitrified at the 8-cell and re-vitrified at the compact stage, Group 5; Vitrified at the 8-cell stage and re-vitrified at the early blastocyst stage, and a; Significant difference with group 1 (P<0.05).

## Discussion

The present research evaluated the impact of revitrification on developmental rate and profile of apoptotic gene expressions in compact and early blastocyst stage embryos. The results showed that embryos after vitrification had a similar in vitro developmental rate to fresh embryos. However, re-vitrified embryos had a reduced rate compared to fresh embryos. In contrast to the current study results, other studies reported that re-vitrification did not negatively affect survival and developmental potential (16-18).

The differences between these reports and our observationsmight be due to formulation of different VS, embryo quality, species, vitrification method and culture time between vitrification and re-vitrification, and addition of antioxidants to the VS. Ito et al. (16) evaluated the impact of embryo re-vitrification (2-cell, 4-cell, morula, and blastocyst) on the same stage on embryo developmental competence. They reported that re-vitrifaction with the cryotop did not affect the developmental ability of mouse embryos. One reason for this disparity might be the stage of embryos during the re-vitrification time. The current study vitrified 8-cell embryos that were cultured in HTF medium until the morula and early blastocyst stages. Hence, re-vitrification was performed at a later stage.

Sheehan et al. (17) re-vitrified mouse embryos at a different stage and reported that re-vitrification did not affect survival and degeneration rates of the embryos. They added ascorbate to the VS as an antioxidant. The addition of an antioxidant might improve survival rate. El- Gayar et al. (19) demonstrated that the in vitro and in vivo developmental rates of mouse blastocysts vitrified once or twice were similar to fresh embryos. However, a significant proportion of embryos could not tolerate vitrification for a third time. Vitale et al. (20) repeated freezing mouse embryos without a culture time intervention between each cycle after thawing contained lower mean cell numbers at hatching compared to unfrozen embryos. This might indicate that the embryos experienced some stress during successive vitrification procedures.

It has been shown that during cooling and warming attributed to thermal shock, the cytoskeleton endures reversible or irreversible changes (21). Mozdarani and Moradi (22) reported that vitrification in solution with EG could cause chromosomal aberrations and reduced embryo viability in vitro. Exposure of oocytes and embryos to a concentrated solution of cryoprotectants during the vitriﬁcation process could have caused an increase in cytoskeletal disruption and abnormal spindles, which resulted in poor survivability and developmental capacity (23). Rho et al. (24) reported that damage to microtubules and mitochondria during oocyte cryopreservation might be involved in the reduced viability. The adverse effect of cryopreservation might lead to the formation of cracks in the zona pellucida, or damages to the cell membranes and intracellular components (25, 26). Mohr and Trounson (27) concluded that cryoinjury to embryos might be selective for one cell type within an embryo, and the extent and nature of damage depended on the developmental stage. Several publications have postulated that the developmental stage of an embryo during vitrification had an important role in the outcome after the vitrification process.

The developmental stage is believed to have an important role in successful re-vitrification (7, 8). We re-vitrified embryos at different developmental stages. We chose compact and early blastocyst stage embryos to assay the effect of embryo stage during the re-vitrification process on embryo developmental rate. Our findings showed that re-vitrification in both the early blastocyst and compact stages had similar effects on in vitro developmental rate. We have observed that both groups of embryos had reduced developmental rates.

The survivability of oocytes and embryos following cryopreservation procedures depends on different mechanisms of cell injury such as the chemical toxicity of the cryoprotectants, osmotic shock in the dehydration and rehydration procedures, and vitrification effects on the ultrastructure of oocytes and embryos (23). Previous studies have shown that cryoprotectants such as DMSO and PrOH could affect microtubule organization and distribution of mitochondria in several species, including mice and humans (28, 29). Mitochondria played an important role in Ca^2+^ signaling that mediated oocyte activation and development, and apoptotic cell death (30). Disruption of mitochondria increased cell permeability to Ca^2+^ and caused increased intracellular Ca^2+^ thus triggering hydrolytic enzyme activity, impaired energy production, and cell death (31).

We have evaluated the possible impact of revitrification on *Bax* and *Bcl-2* (apoptotic) gene expressions. Apoptosis is a regulated program that initiates cell death. Members of the Bcl-2 family have an important role in control of apoptosis. Our findings have shown that re-vitrification in both the compact and early blastocyst stages could alter expressions of *Bax* and *Bcl-2* genes. Re-vitrification upregulated *Bax* and downregulated *Bcl-2*. *Bax* is pro-apoptotic and induces cell death whereas *Bcl-2* is anti-apoptotic and promotes survival (32). Therefore, the decreased developmental capability of re-vitrified embryos has a relation to regulation of apoptosis pathways.

The results of a study by Dhali et al. (33) showed a strong relationship between compromised developmental competence and altered transcriptional activities of *Bax*, *Bcl-2*, and *p53* in treated embryos. In the present study, we observed a relation between decreased developmental rates a change in apoptotic gene expressions. However, the apoptotic gene expression profiles were similar between two re-vitrified groups.

## Conclusion

We have shown that re-vitrification during compact or blastocyst stages had the same effect on developmental potential. Re-vitrification could alter apoptotic gene expressions. However it is necessary to study additional genes involved in apoptosis regulation.
